# More Than Meets the Eye: A Case of Breast Cancer Switching from Being Luminal-Androgen-Receptor-Positive to Being Hormone-Receptor-Positive

**DOI:** 10.3390/medicina59101875

**Published:** 2023-10-22

**Authors:** Federica Martorana, Giuseppe Di Grazia, Giovanni Nunzio Rosano, Giada Maria Vecchio, Chiara Conti, Sabrina Nucera, Gaetano Magro, Paolo Vigneri

**Affiliations:** 1 Department of Clinical and Experimental Medicine, University of Catania, 95123 Catania, Italy; paolo.vigneri@unict.it; 2 Department of Human Pathology “G. Barresi”, University of Messina, 98131 Messina, Italy; giuseppedigrazia25@gmail.com (G.D.G.); conchiara9@gmail.com (C.C.); sabrinanuc@libero.it (S.N.); 3 Department of Medical, Surgical Sciences and Advanced Technologies “G.F. Ingrassia”, Anatomic Pathology, University of Catania, 95123 Catania, Italy; giovanninunziorosano@gmail.com (G.N.R.); giadamariavecchio@gmail.com (G.M.V.); gmagro@unict.it (G.M.); 4 Humanitas Istituto Clinico Catanese, University Oncology Department, 95045 Catania, Italy

**Keywords:** breast cancer, triple-negative breast cancer, luminal androgen receptor, molecular testing, PIK3CA, everolimus

## Abstract

Triple-negative breast cancer (TNBC) represents about 15% of all breast cancers and is usually characterized by aggressive clinical behavior and a poor prognosis. Four TNBC subgroups have been previously defined with different molecular profiles: (i) luminal androgen receptor (LAR), (ii) mesenchymal (MES), (iii) basal-like immunosuppressed (BLIS) and (iv) basal-like immune-activated (BLIA). Among these, LAR is characterized by the expression of the androgen receptor (AR), and exhibits genomic characteristics that resemble luminal breast cancers, with a still undefined prognosis and clinical behavior. Here, we report a case of a woman affected by recurring LAR TNBC, which underwent phenotypic changes throughout its natural history. After the initial diagnosis of LAR breast cancer, the patient experienced local recurrence with strong expression of the estrogen receptor. Due to this finding, she started treatment with a CDK4/6-inhibitor and an aromatase inhibitor, followed by oral vinorelbine, both with dismal outcomes. Then, she received everolimus and exemestane, which determined temporary disease stabilization. An extensive NGS analysis of tumor tissue showed *PIK3CA* and *HER2* mutations. Our case is consistent with previous reports of LAR breast cancer and underlines the potential utility of re-biopsy and molecular testing in breast cancer (BC), especially in rare subtypes.

## 1. Introduction

Triple-negative breast cancer (TNBC) is defined by the lack of hormone receptors (HR) and epidermal growth factor receptor 2 (HER2) overexpression or amplification [1]. TNBC accounts for 15% of all breast cancers and is usually characterized by aggressive clinical behavior and a poor prognosis [2,3]. Recently, the introduction of next-generation sequencing (NGS) and *omics* technologies have defined several biological features of TNBC, showing considerable disease heterogeneity [4,5]. Indeed, TNBCs may be characterized by either a limited number of somatic mutations or by a high number of genetic alterations in several signaling pathways [6,7]. Based on their molecular profiles, chemosensitivity and presence of potential therapeutic targets, six TNBC subtypes have been identified by Lehmann and colleagues: basal-like 1, basal-like 2, immunomodulatory, mesenchymal, mesenchymal stem-like and luminal androgen receptor (LAR) [8]. Likewise, Burstein et al. have described four TNBC variants characterized with distinct molecular profiles: (i) luminal androgen receptor (LAR), (ii) mesenchymal (MES), (iii) basal-like immunosuppressed (BLIS) and (iv) basal-like immune-activated (BLIA) [9].

Among these subtypes, LAR is characterized by the expression of the androgen receptor (AR) and usually displays apocrine histological features. These tumors exhibit a gene expression profile that resembles luminal breast cancers (e.g., *FOXA1*, *GATA3*, *SPDEF* and *XBP1* hyperexpression) and are usually enriched in *PIK3CA*, *KMTC*, *CDH*, *NF1*, and *AKT1* mutations [8,10,11,12]. However, the prognosis and clinical behavior of LAR breast tumors remain undefined, with conflicting outcomes emerging from the available literature [13,14,15,16]. Similarly, the role of the AR in predicting responses to anti-androgen therapies is unclear, with conflicting results from clinical trials.

Here, we report a case of recurring LAR TNBC, which presents phenotypic changes throughout its natural history.

## 2. Detailed Case Description

**Figure 1:**
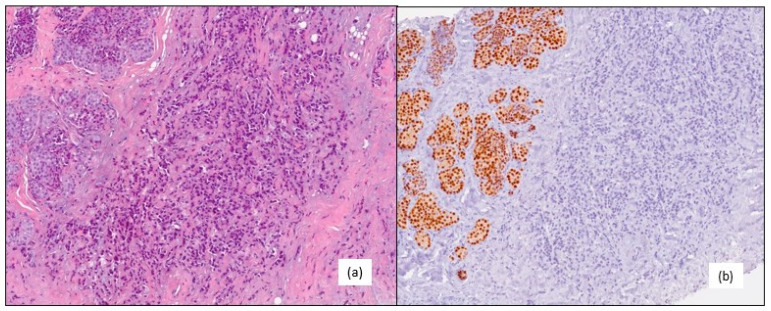
(**a**) Classic invasive lobular carcinoma (G2) hematoxylin–eosin 10×. (**b**) Negative estrogen receptors in invasive lobular carcinoma; positive internal control in lobular intraepithelial neoplasia, immunohistochemistry 10×.

A 61-year-old post-menopausal woman was admitted to our hospital in February 2015 for a right breast lump. A core needle biopsy revealed an invasive lobular breast carcinoma, grade 2, with a clinical stage of T1c N0, which tested negative for the estrogen (ER) and progesterone receptors (PgR), and AR 70%; HER2 1+ on immunohistochemistry (IHC), with a Ki67 proliferation index <1% (Figure 1).

No family history of breast cancer was reported. She had a personal history of congenital renal hypoplasia and had been receiving dialytic treatments since 2007. Tumor staging through abdominal ultrasonography, chest X-ray, and bone scan did not show distant metastases. Consequently, the patient underwent a right mastectomy and sentinel lymph-node biopsy with a final histopathological exam showing a pT1c pN0 (positive for isolated tumor cells [i+]) M0 TNBC, negative ER and PgR, AR 60%, HER2 1+, and a Ki67 < 1%. After surgery, the patient began regular follow-up evaluations.

**Figure 2:**
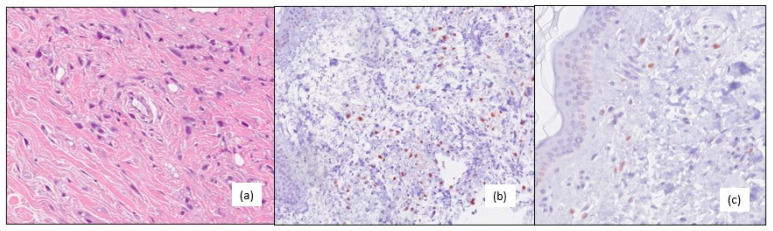
(**a**) Dermic pleomorphic invasive lobular carcinoma (G3), hematoxylin–eosin 20×. (**b**) Positive androgen receptors, immunohistochemistry 20×. (**c**) Positive estrogen receptors, immunohistochemistry 20×.

In October 2016, she underwent surgical excision for a local recurrence on her post-mastectomy surgical scar with a diagnosis of grade 3, negative ER and PgR, AR positive (60%), HER2 IHC 0 and a Ki67 1% lobular invasive breast carcinoma (Figure 2). During this time, chest wall irradiation was performed and the patient resumed follow-up.

**Figure 3:**
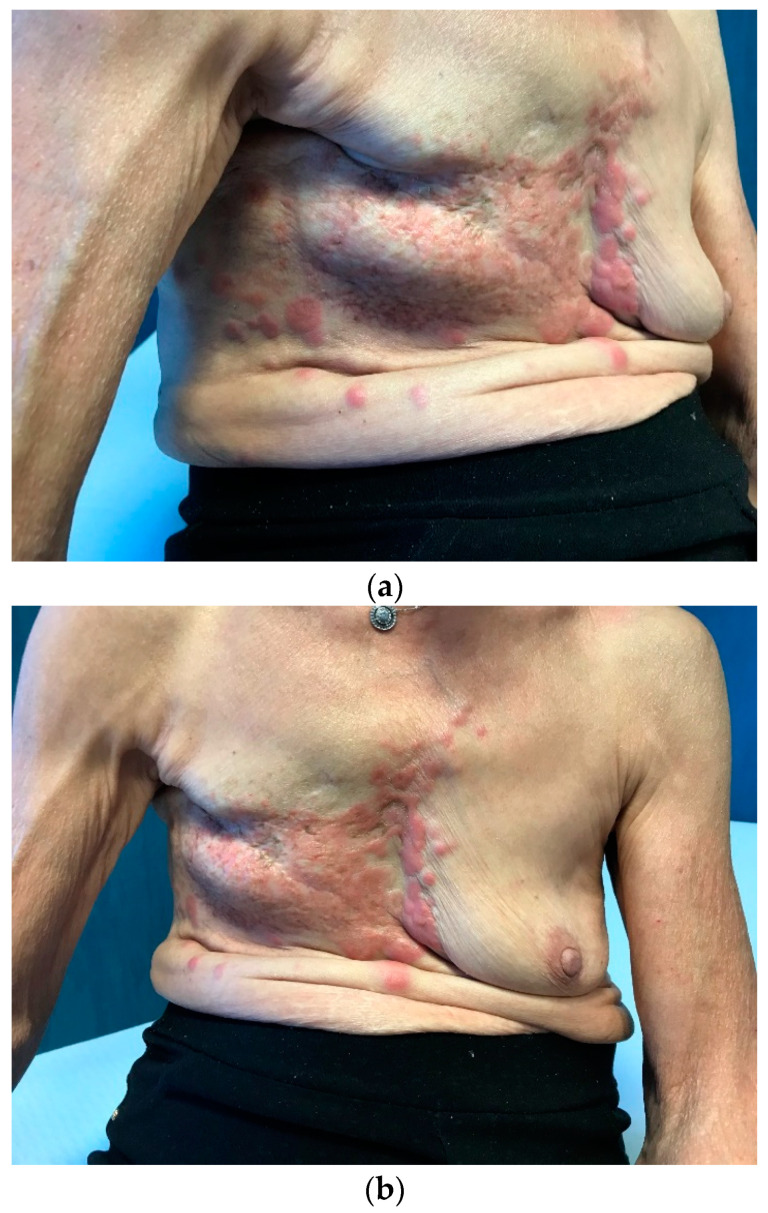

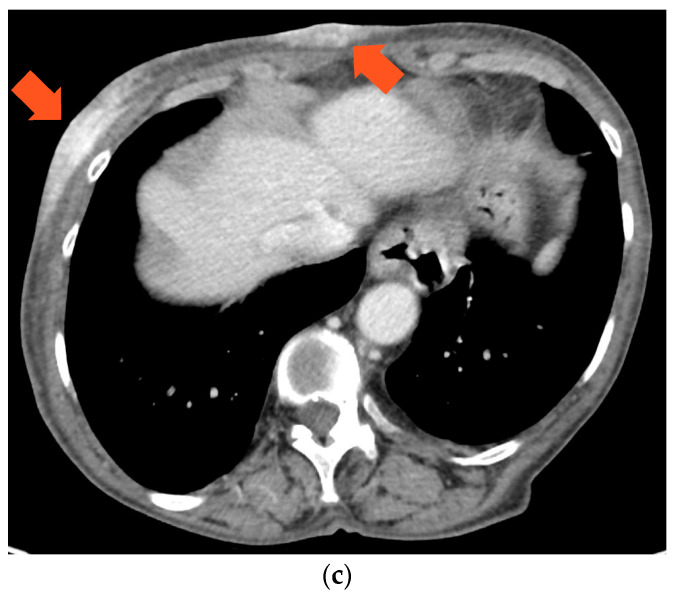
(**a**,**b**) Chest wall recurrence and (**c**) tumor cutaneous infiltration at the CT scan.

In February 2021, the patient returned to us due to the onset of multiple cutaneous erythematous nodules on the chest wall, which were consistent with a new recurrence of pleiomorphic lobular breast carcinoma, grade 3 (Figure 3). A core-needle biopsy revealed that the tumor had acquired endocrine sensitivity, with ER 70%, negative PgR expression, AR 70%, HER2 IHC 1+, and Ki67 10%. Carcinoembryonic antigen (CEA) and carbohydrate antigen 15.3 (CA15.3) were slightly elevated, namely 6.1 ng/mL (range 0–5) and 35.2 IU/mL (range 0–27), respectively. A total body contrast-enhanced computed tomography (CT) scan was performed, which excluded the presence of distant metastases (Figure 3C). Given the HR positivity, the patient started standard first-line therapy with the cyclin-dependent kinase 4/6 inhibitor (CDK4/6i) palbociclib and letrozole.

**Figure 4:**
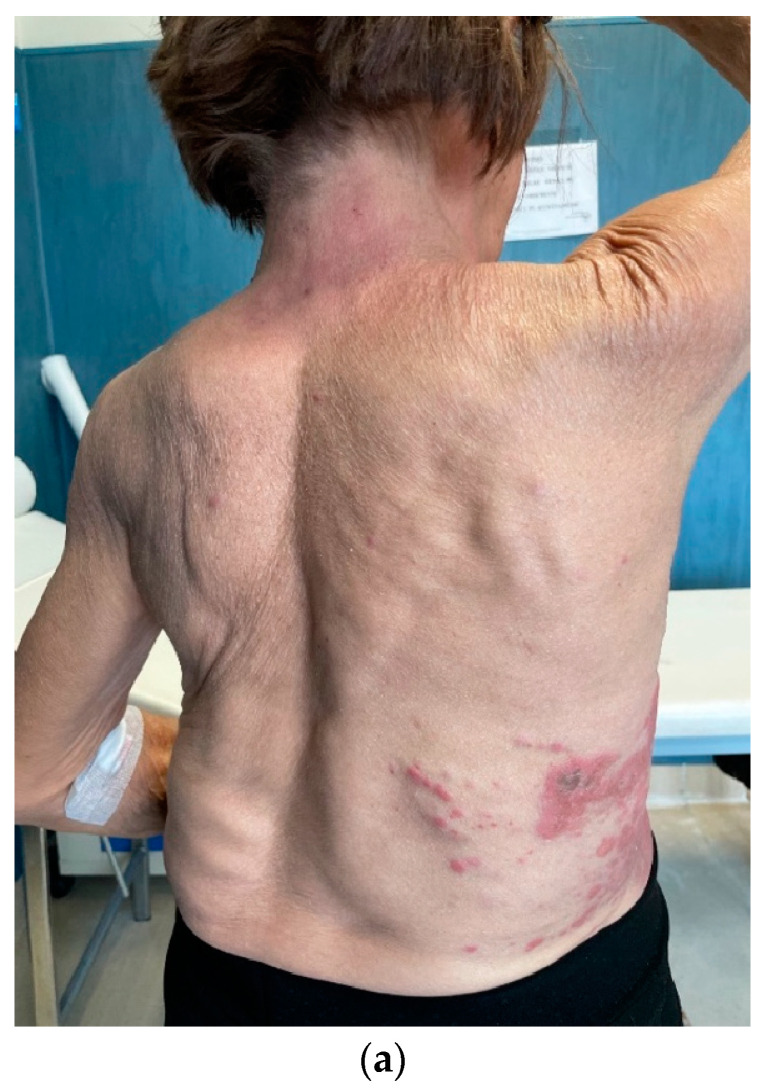

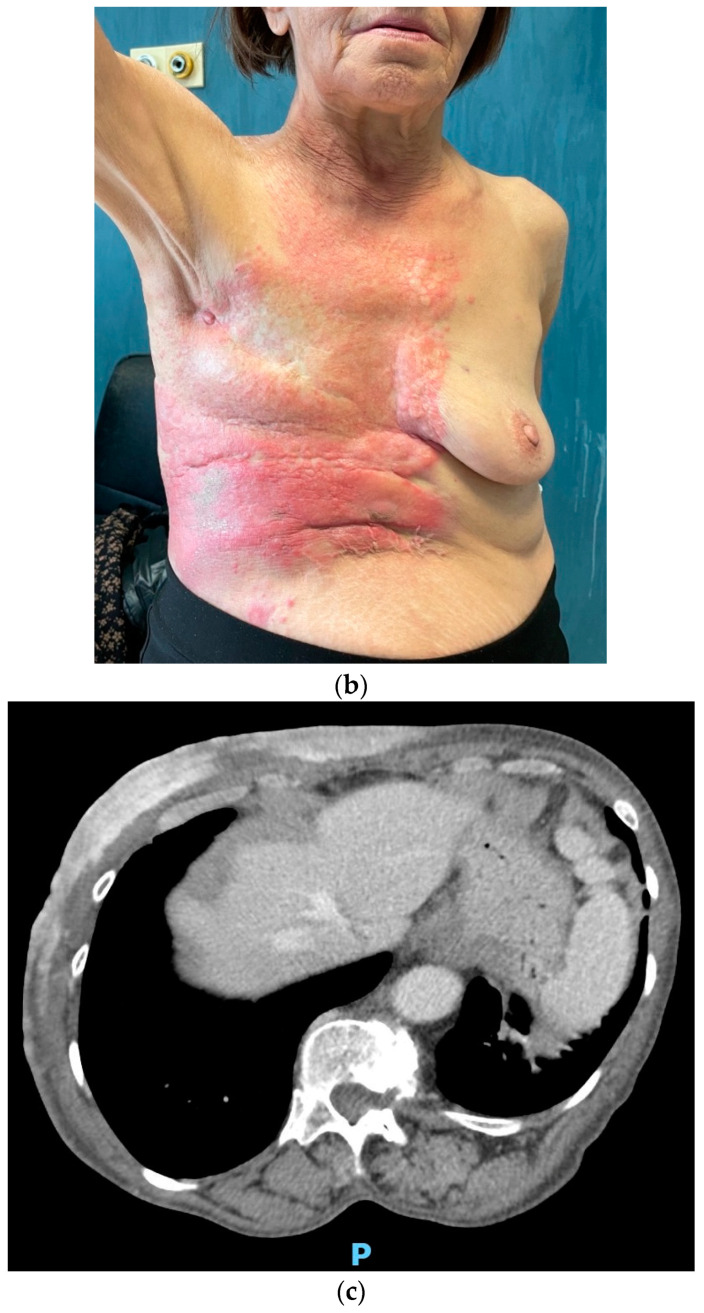
(**a**,**b**) Clinical and (**c**) radiological disease progression in November 2021.

After experiencing initial disease stability with decreasing tumor markers (CEA 2.1 and CA15.3 22.3 IU/mL), in November 2021, unequivocal cutaneous progression occurred, with ulceration and spreading of the erythematous lesions to the upper abdomen (Figure 4). CEA remained below the upper limit of normal, while CA15.3 increased to 80 IU/mL.

**Figure 5:**
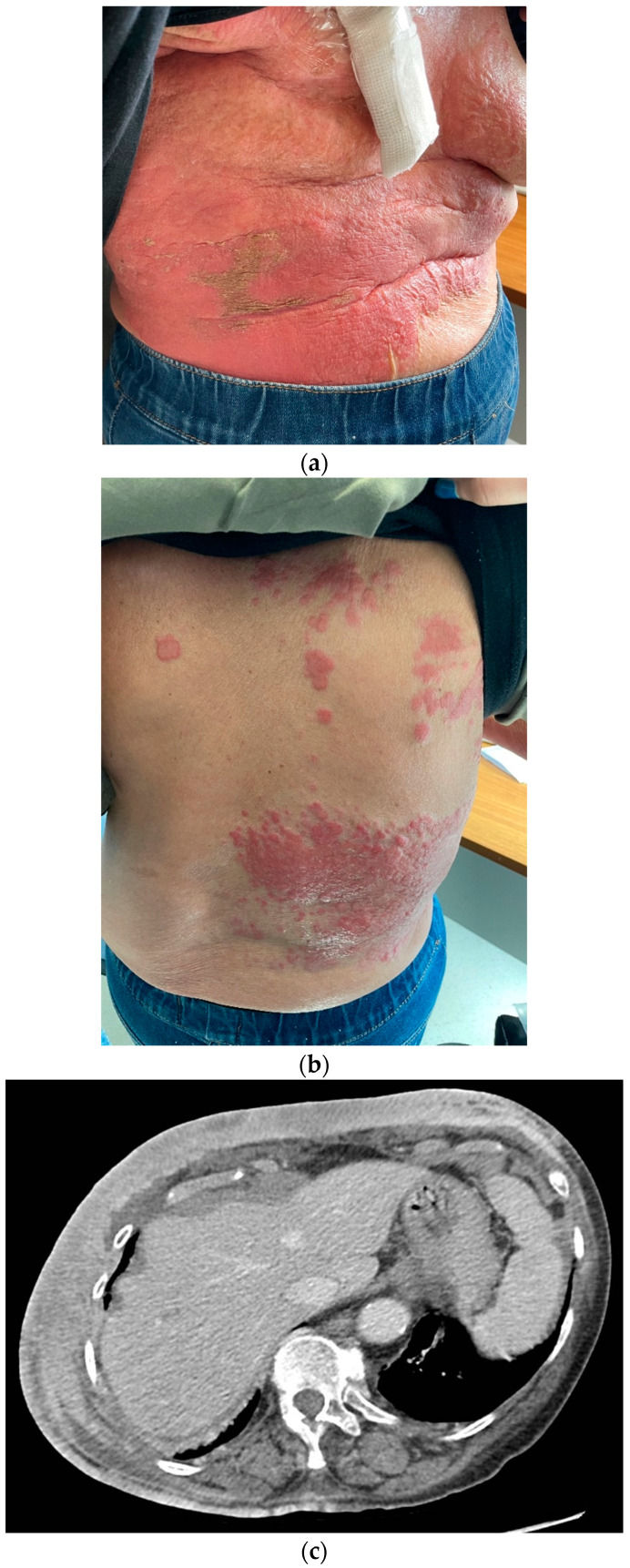

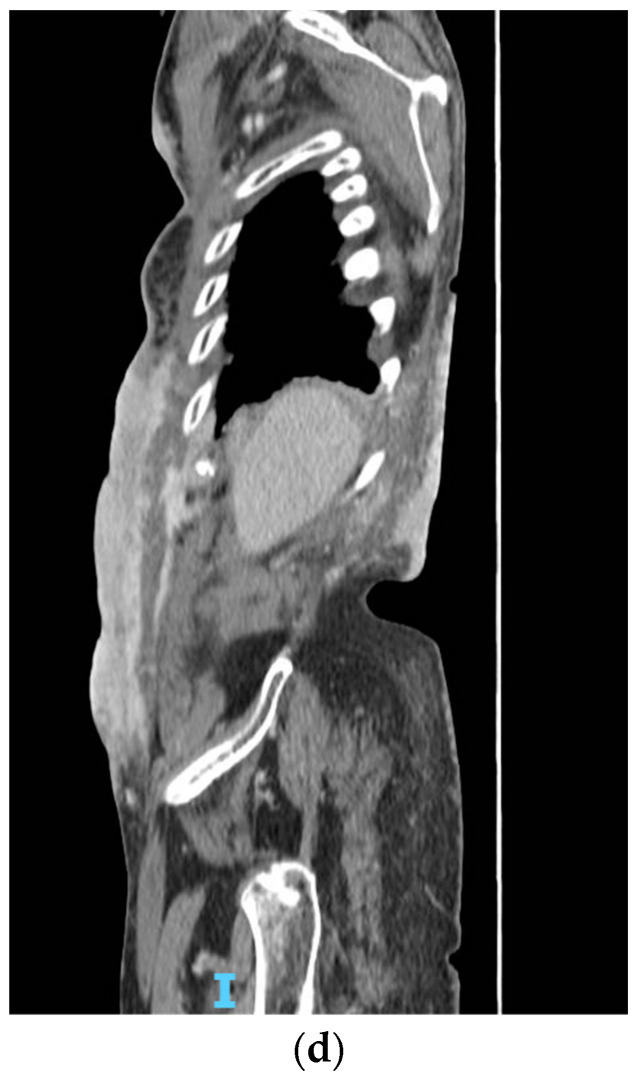
(**a**,**b**) Clinical and (**c**,**d**) radiological disease progression in May 2022.

Considering the scarce endocrine sensitivity and taking the patient’s renal function into account, a second-line chemotherapy with metronomic oral vinorelbine at 30 mg three days per week was initiated. The treatment was poorly tolerated, with recurrent dose interruptions due to hematological toxicity, requiring weekly erythropoietin support. Carbohydrate antigen 15.3 increased to 180.6 IU/mL in March and to 216.7 IU/mL in May 2022. A contrast-enhanced CT scan was then performed, showing worsening of the neoplastic panniculitis, which had widely spread to the lower abdomen, reaching the pubis (Figure 5).

A new skin biopsy was then performed to reassess receptor status. ER and PgR were negative, AR was 70%, while HER2 expression was equivocal (IHC 2+), without gene amplification at fluorescent in situ hybridization (FISH). This result was consistent with the original tumor phenotype and in line with the short response to endocrine-based therapy. Additionally, an internal review of all the tumor’s specimens obtained over time confirmed previous histological diagnoses. A molecular analysis performed on the primary tumor showed a coherent genetic profile (as detailed below). Thus, we assumed that the disease, which had initially switched to a luminal-like subtype, was able to revert to the original LAR-TNBC phenotype as a result of therapy-induced selective pressure.

Despite the lack of HR expression at the last biopsy, we opted for the combination of everolimus and exemestane, given the presence of *PIK3CA* and *HER2* mutations in the molecular analysis (as detailed below), in order to target the original biological driver. In June 2022, before the new line of therapy, CA15.3 was 742,1 IU/mL, while CEA, normal until then, began to increase, with a value of 14.1 ng/mL. This treatment is currently ongoing with cutaneous disease stabilization and a substantial decrease in tumor markers (CEA 10.4 ng/mL and CA15.3 540.2 IU/mL).

## 3. Molecular Analysis

We performed an extensive NGS analysis using a commercially available panel (FoundationOne^TM^, Foundation Medicine Inc., Cambridge, MA, USA) on the primary tumor sample, looking for potentially druggable somatic alterations. The results of the analysis were consistent with pre-existing evidence, revealing a mutation in *PIK3CA* (H1047R) and in *CDH1* (splice site 824_832+17del26) with variances of allele frequency (VAFs) of 11.7% and 11.9%, respectively. A somatic alteration in *ERBB2* (S653C) with a VAF of 3.9% was also present. Microsatellite status was stable (MSS), and tumor mutation burden (TMB) was low. Taken together, a low proliferative and luminal gene expression profile was consistent with the disease’s clinical behavior.

## 4. Materials and Methods

For the immunohistochemical analyses, all biopsy samples had been fixed in 10% neutral buffered formalin for 12 h and embedded in paraffin. Then, 4 µm thick sections were cut and stained with hematoxylin and eosin (H&E) to perform histological diagnosis. Immunohistochemical analyses were performed using the standard streptavidin–biotin-labeling technique using the LSAB kit (Dako, Glostrup, Denmark) with appropriate positive and negative controls. Sections derived from paraffin-embedded specimens were deparaffinized in xylene for 15 min, rehydrated, and treated with 3% H_2_O_2_ for 10 min to block endogenous peroxidase activity, followed by extensive rinsing in double-distilled water and further rinsing for 15 min in 0.01 M phosphate-buffered saline (PBS), pH 7.4. Immunohistochemical studies were performed, testing for the following antibodies: estrogen receptors (ERs), androgen receptors (ARs), progesterone receptors (PgRs), HER2/neu, and nuclear proliferative index/Ki67.

For the next-generation sequencing analysis, nucleic acids were isolated from FFPE samples containing ≥50% tumor cells. Comprehensive genomic profiling was performed using a hybrid capture-based platform (FoundationOne™, Foundation Medicine Inc., Cambridge, MA, USA), which identifies single nucleotide substitutions (SNV), insertions and deletions (indels), copy number alterations (CNAs), and rearrangements [7]. The platform interrogates the coding sequence of 315 cancer-related genes and introns from 28 genes often rearranged in solid tumors to a median depth of coverage greater than 500× [17].

## 5. Discussion

Phenotype discordance in hormone receptors and HER2 status between primary and recurrent breast cancer is not a rare event in the natural history of the disease [18]. This phenomenon seems to be related to several factors, rather than a single mechanism. Variability in the accuracy and protocols of IHC staining may alter the analyses, leading to false-negative or false-positive results. Moreover, even when the analytical process is standardized, it may lead to discordant results, due to suboptimal reproducibility [19]. For these reasons, it should be standard practice to perform an internal review of all the tumor’s specimens when discordance is detected. In addition to analytical errors in receptor assessment, intratumor heterogeneity and phenotypic changes may provide a biological explanation. In a meta-analysis, Aurilio et al. estimated a discordance rate for ER, PgR and HER2 of 20%, 33% and 8%, respectively [20]. Moreover, recent studies with NGS reinforced the hypothesis that variation in ER, PgR and HER2 status may actually reflect clonal evolution. The reasons behind tumor heterogeneity may be a consequence of biological drift, therapy-dependent selective pressure causing clonal selection or the contemporary presence of small sub-clones undetected within the primary tumor. From a literature review, PgR is the parameter more frequently discordant. Its loss seems to be associated with lower overall survival, due to a hypothetic protective role of this receptor against metastatic spread [21]. Additionally, ER loss in metastatic sites may be associated with a worse prognosis and reduced overall survival [19,22]. Acquisition of HER2 expression in metastatic tissue is a known mechanism of endocrine resistance and leads to an increase in disease aggressiveness, yet it does not affect survival given the effective therapies available [19]. On the other hand, HER2 loss may worsen prognosis. However, evidence is scarce as it represents a rare event in breast cancer [19]. Overall, the evidence is not solid enough to assess a concrete prognostic value for phenotypic discordance, and further studies are needed. For the abovementioned reasons, tumor re-biopsy at progression is usually advisable, when feasible and safe, either to confirm metastasis diagnosis or assess biological features of the tumor in order to consider new treatment options.

The presented case is an example of tumor’s phenotype variability. By recognizing these changes, we tried to tailor therapeutic choices to the evolving landscape of the disease. Despite these efforts, the described breast malignancy was scarcely sensitive to our initial therapies.

To better understand the disease, we performed genetic profiling that showed a low proliferative and luminal gene expression profile, consistent with the LAR phenotype. Indeed, AR expression has been regarded as a potential therapeutic target for TNBC. However, despite showing promising pre-clinical activity, trials testing anti-androgen monotherapy in LAR BC failed to show substantial benefit [23,24,25,26]. Bicalutamide, an anti-androgen agent, and abiraterone acetate, a 17-[α]-hydroxylase/17,20-lyase (CYP17) inhibitor, were not able to significantly improve the median progression-free survival (mPFS) and overall response rates (ORRs) in patients with advanced LAR BC in phase II clinical trials [24,25,26]. In the same setting, darolutamide, another anti-androgen agent, failed to ameliorate the clinical benefit rate (24.5% versus 47.8%) and mPFS (1.8 months versus 3.6 months) when compared to capecitabine [27]. Studies are ongoing to test combinations of anti-androgens and other drugs, such as CDK4/6i or PIK3CA inhibitors [28,29]. It would be of interest to evaluate combinations of other inhibitors of the same pathway, such as AKT inhibitors, which already showed activity in ER-positive breast cancer and will probably enter clinical practice in the forthcoming future [30]. Since our patient’s disease is enriched with PI3K-AKT-mTOR pathway alterations, monotherapy or combination therapy of these agents may represent an option in the future [31,32]. While our patient was not eligible for enrolment in any clinical trial due to her kidney disease, other subjects with similar tumor characteristics may gain early access to molecularly driven therapies in the context of a clinical trial.

## 6. Conclusions

In conclusion, this case is consistent with pre-existing evidence concerning the clinical behavior and biological characteristics of LAR TNBC, which represents a challenging disease. Moreover, it confirms the potential utility of re-biopsy and molecular testing to track tumor evolution over time, especially in cases lacking therapeutic targets. Genomic analyses may shed light on alterations involved in disease development and progression, providing evidence for molecularly driven decision-making.

## Data Availability

All data generated or analyzed for this report are included in the published article.
